# Early Outcomes After the Adoption of the Direct Anterior Approach for Total Hip Arthroplasty

**DOI:** 10.7759/cureus.80417

**Published:** 2025-03-11

**Authors:** Joshua D Rutnagur, Luke Farrow, Iain A Rankin, Karola Pawloy, Christopher Munro

**Affiliations:** 1 Department of Trauma and Orthopaedics, NHS Education for Scotland, Aberdeen, GBR; 2 Department of Trauma and Orthopaedics, NHS Grampian, Aberdeen, GBR; 3 Department of Trauma and Orthopaedics, Woodend Hospital, Aberdeen, GBR

**Keywords:** anterior, arthroplasty, hip, oxford, posterior

## Abstract

Background and objective

Our unit had historically performed total hip arthroplasty (THA) through either the posterior (PA) approach or the anterolateral approach (ALA). In November 2020, a group of five consultants transitioned to utilising the direct anterior approach (DAA). Appropriate training was undertaken, and cases were performed as dual consultant procedures with intraoperative radiography or robotic assistance. In this study, we aimed to determine if the learning curve of adopting the DAA approach had led to adverse outcomes when compared to the previously used PA or ALA.

Methodology

Outcomes were collated prospectively. These included basic demographics, intraoperative details, complication rates, and Oxford Hip Scores (OHS). The primary outcome measure was OHS. A comparison of outcomes was made across three patient groups who underwent THA either via DAA, PA, or ALA. All patients were operated on between November 2020 and September 2021.

Results

A total of 48 patients underwent DAA THA over one year. The mean age was 67 years and the mean American Society of Anaesthesiologists (ASA) score was 2. Over this period, 140 PA and 137 ALA THAs were performed with available data. Propensity score matching was performed on a 1:1 basis using BMI, age, sex, and ASA scores as covariates to generate a matched cohort group of conventional-approach THA (n=32). Length of stay was significantly reduced at 2.2 days (p<0.001) with DAA compared to ALA and PA. However, there was no significant difference in the length of surgery, blood loss, infection, dislocation, and periprosthetic fracture rate. There was no significant difference in OHS between any approach at three months or one year.

Conclusions

The transition to the DAA approach has not made a negative impact despite its associated steep learning curve and, in fact, has improved efficiency in elective surgery. Based on our findings, we recommend that those shifting to this approach receive the appropriate training in a high-volume centre and perform cases as dual consultant procedures.

## Introduction

Total hip arthroplasty (THA) is an established and effective treatment for hip diseases such as degenerative and inflammatory arthritis, femoral neck fracture, avascular necrosis, and hip dysplasia [1]. The procedure has been proven to significantly relieve symptoms of pain, improve stiffness, and restore function and quality of life [2]. Historically, various surgical approaches have been used to perform this operation, including the anterolateral approach (ALA), the direct anterior approach (DAA), and the posterior approach (PA), which is the most widely used globally [3]. Similar to many other units, our arthroplasty unit (at a major tertiary referral centre in Scotland) historically performed THA via the ALA or PA approaches. In October 2020 a group of five arthroplasty surgeons transitioned to using the DAA. Appropriate training was undertaken, and cases were performed as dual consultant procedures with intraoperative X-ray or robotic assistance.

This study aimed to compare outcomes in patients who underwent THA) utilising the DAA to those in patients who underwent PA or anterolateral approach ALA for THA, after the adoption of the DAA at our unit over one year. Outcomes data were collected prospectively. Surgeons have highlighted the learning curve as the major barrier to adopting DAA when compared to other approaches. Currently, there is a lack of evidence comparing the outcomes of the learning curve of DAA and other approaches, which this study aimed to address.

This article was previously presented as an abstract at the 2021 Winter Scottish Committee for Orthopaedics and Trauma (SCOT) meeting on January 29, 2021.

## Materials and methods

Three hundred and twenty-five patients admitted to our hospital for primary THA between October 2020 and September 2021 were included in this study. A total of 48 patients underwent DAA THA. Over the same period, 140 PA and 137 ALA THAs were performed with available data. Data were collected prospectively and recorded, withholding patient identifiable data, in a database stored on a secure network within NHS Grampian computers. The operations were performed by five arthroplasty consultant surgeons who adopted DAA in October 2020, having previously performed operations via PA or ALA. Operations were performed either using standard THA surgical techniques or with MAKO® robotic or image intensifier assistance.

Data collected included basic demographics, intraoperative details, complications, and Oxford Hip Scores (OHS). Propensity score matching was performed on a 1:1 basis using BMI, age, gender, and American Society of Anaesthesiologists (ASA) score as covariates to generate a matched cohort group of conventional-approach THA (n=32). The primary outcome measure was OHS. Secondary outcome measures included duration of hospital stay, duration of surgery, blood loss, infection, dislocation, and periprosthetic fracture rate. These data were then compared for those who underwent DAA, PA, and ALA and were eligible for inclusion.

Operative data were collected from operation notes prepared by the operating team using the Opera software. Data regarding length of stay was collected from discharge letters prepared by the ward staff using TrakCare® software. Data regarding complications of infection, dislocation, or periprosthetic fracture were collected from documentation from subsequent attendances at the hospital such as clinic visits, acute admissions, or review by the on-call orthopaedic team. This documentation was located on the TrakCare® software. A comparison was made across the three treatment groups using the Kruskal-Wallis Test, comparing OHS at three months and one year, length of hospital stay, and operative blood loss.

Operative technique

Direct Anterior Approach

The patient is positioned supine on a fluoroscopy table, utlising an approximately 10 cm skin longitudinal incision centred over the lateral border of the greater trochanter (GT). This is made in a line drawn from the anterior superior iliac spine (ASIS) to the lateral femoral condyle. The internervous plane is between the superior gluteal nerve supplying tensor fascia lata, and the femoral nerve supplying rectus femoris. The lateral femoral cutaneous nerve is located in the medial portion of the approach and protected. The lateral circumflex femoral artery and vein are ligated or coagulated. Gluteus minimus is retracted to expose the anterior capsule, which is repaired at the end of the procedure.

## Results

Propensity score matching yielded a cohort group of 32 patients with a median age of 70.5 years, BMI of 27 kg/m^2,^ and ASA grade 2. A comparative analysis of the groups is presented in Table 1.

**Table 1:** Matched cohort comparison

Surgical approach	Mean length of stay (LoS), days	Mean surgical time, minutes	Mean surgical blood loss, ml	Median Oxford Hip Score at 3 months	Median Oxford Hip Score at 12 months
Anterolateral (ALA)	3.8	101.2	510	37	43.5
Direct anterior (DAA)	2.2	101.2	516	38.25	46
Posterior (PA)	2.9	94.6	513	41	45.5

In the entire cohort of 96 patients, there were two documented postoperative complications. In the DAA group, one of 32 patients had neuropraxia of the lateral femoral cutaneous nerve of the thigh postoperatively, which had completely resolved at three months postop check. In the anterolateral group, one of 32 patients had a documented limp one year postoperatively. The posterior group had no significant complications following THA.

**Figure 1:**
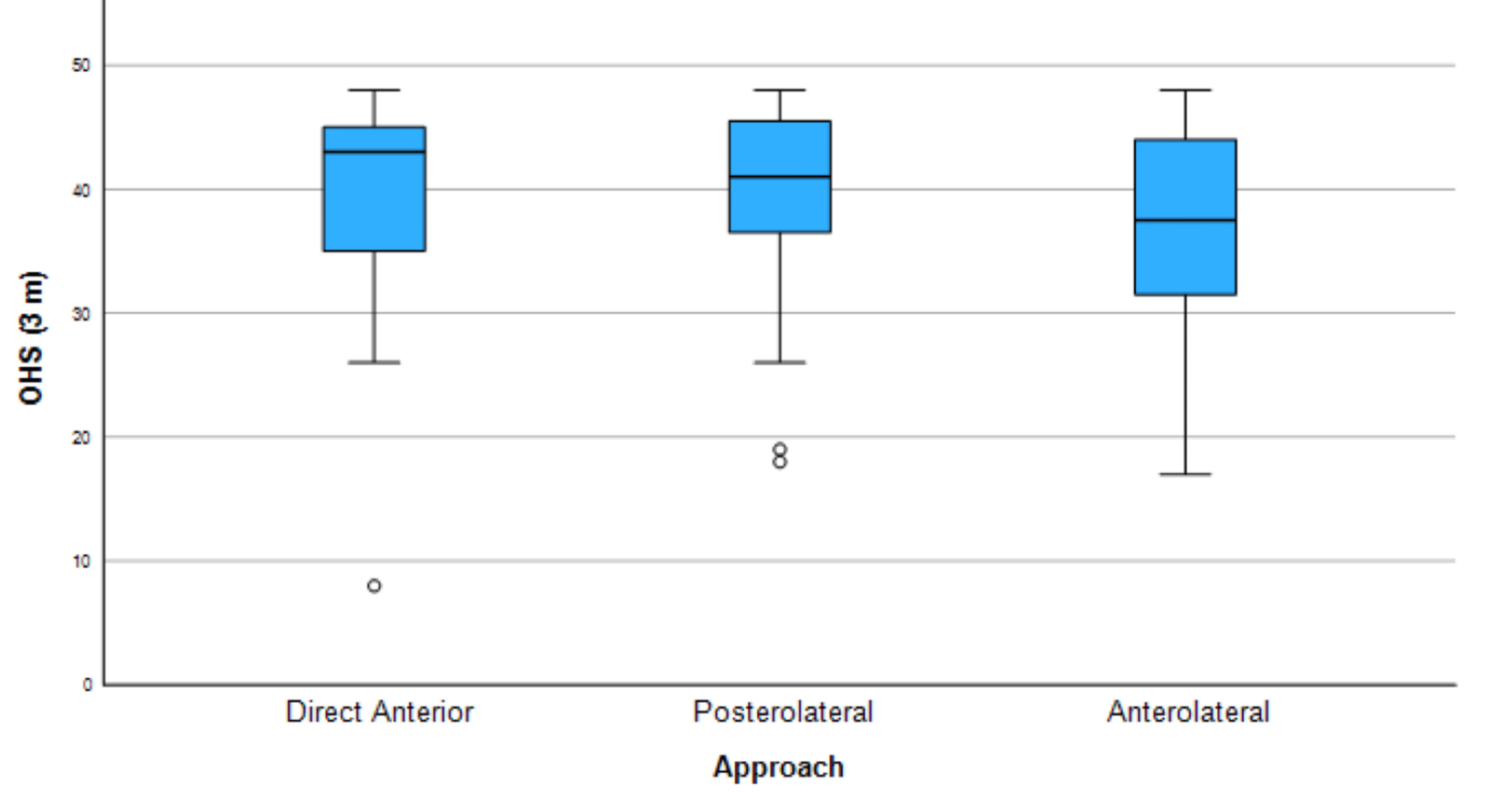
Kruskal-Wallis test summary for OHS at three months OHS: Oxford Hip Score

**Figure 2:**
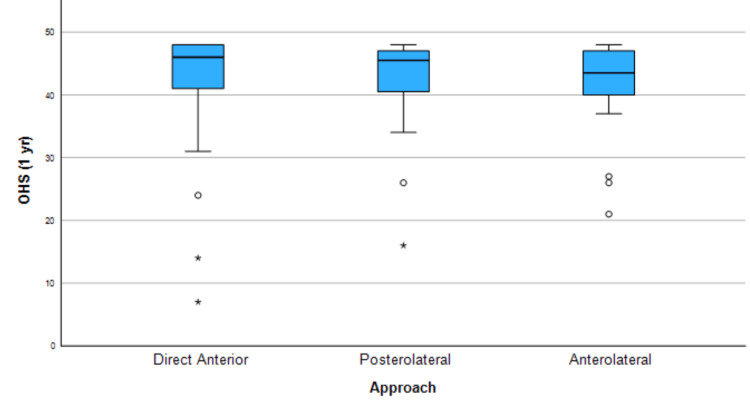
Kruskal-Wallis test summary for OHS at one year OHS: Oxford Hip Score

There was no significant difference across the three patient groups in the primary outcome measure of OHS at three months (a significant value of 0.405). The results are presented below, in Figure 1. Also, no significant difference was found between the three groups in OHS at one year (Figure 2).

**Figure 3:**
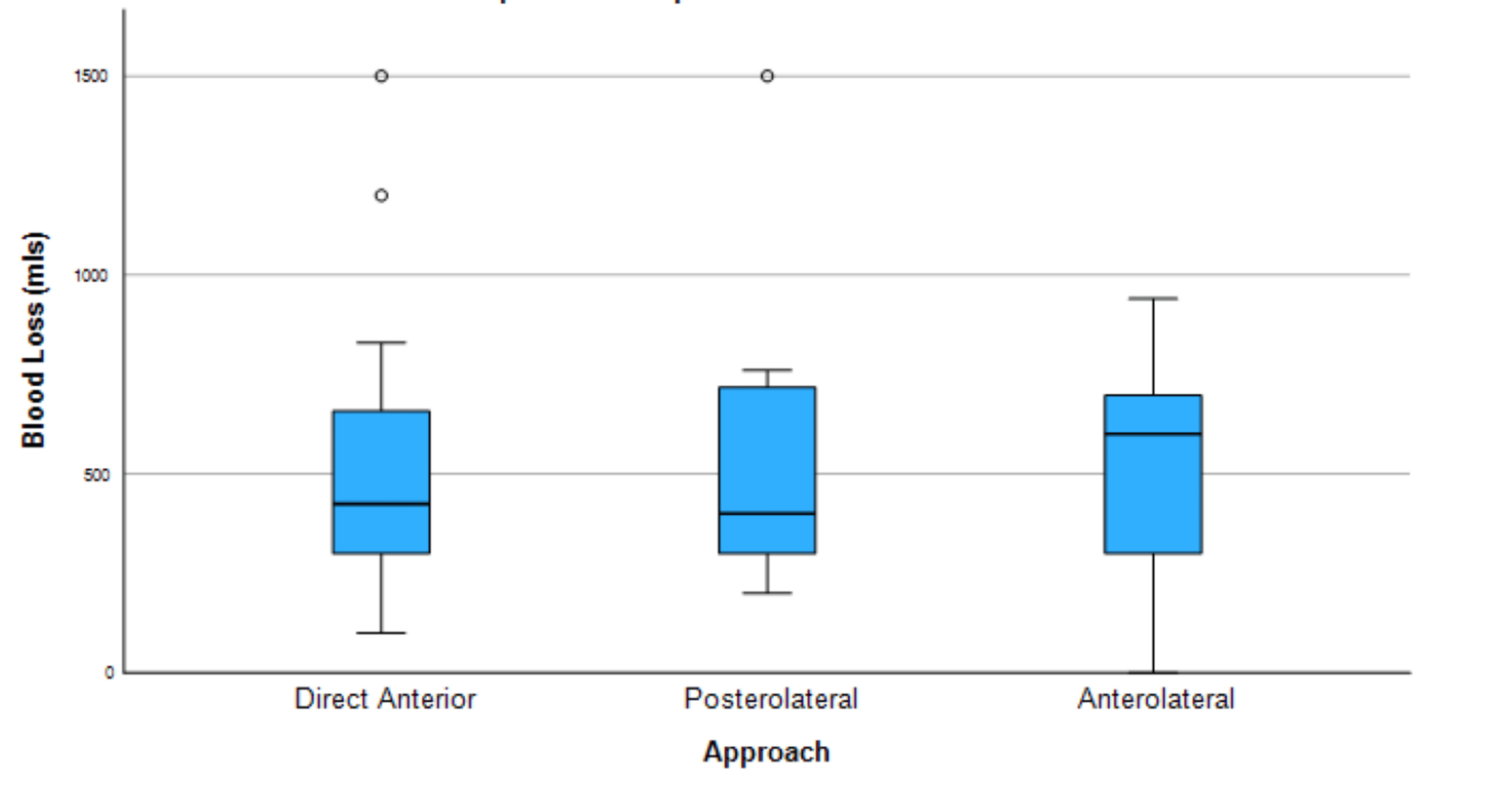
Kruskal-Wallis test summary for operative blood loss

Additionally, no significant difference was found in blood loss between the three different approaches (Figure 3).

**Figure 4:**
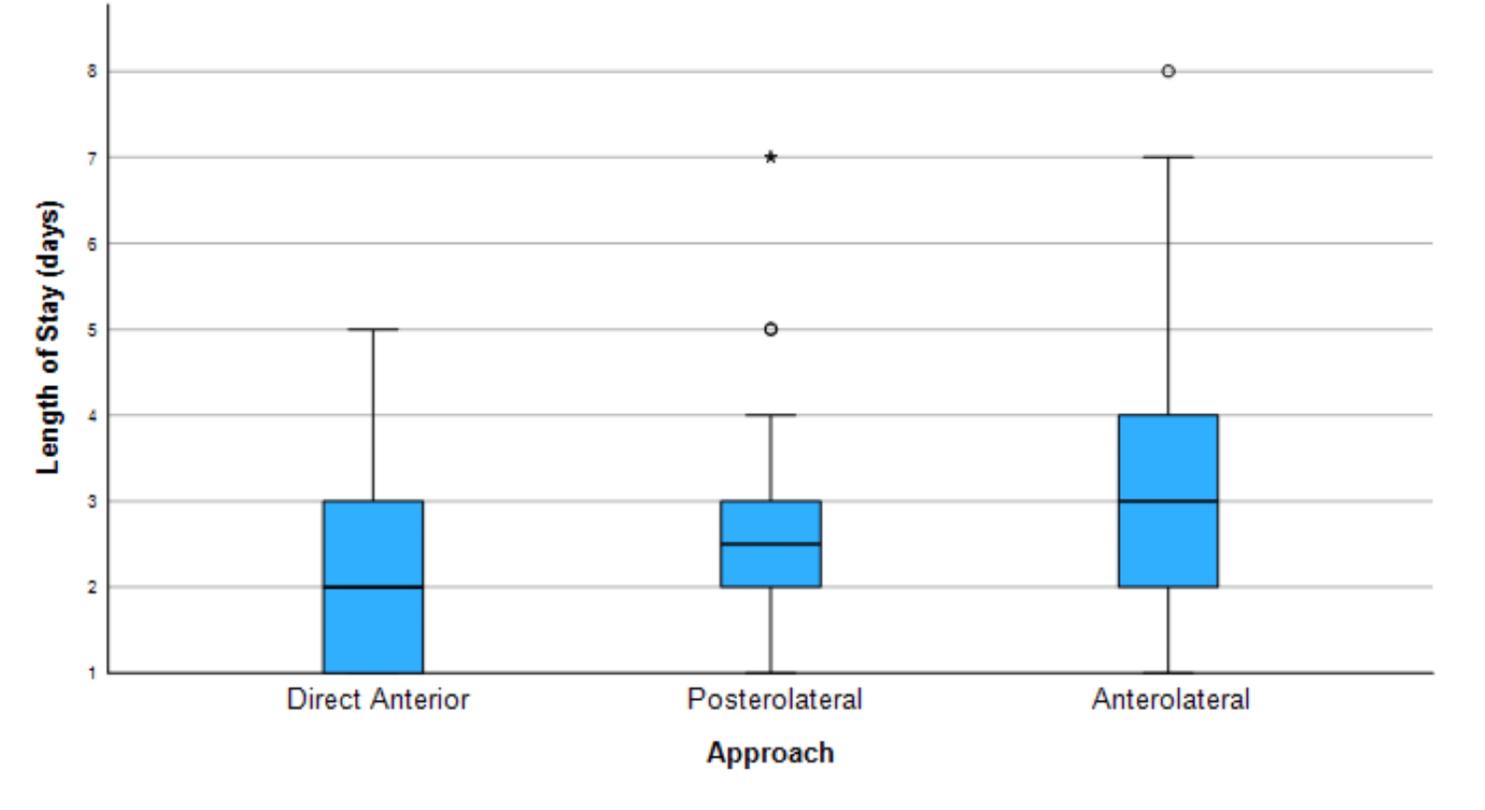
Kruskal-Wallis test summary for length of stay in days

Length of stay was found to be significantly shorter in patients who underwent DAA THA when compared to those who received THA with posterior and anterolateral approaches (a significance value of 0.036), as shown in Figure 4. No significant difference was found in surgical time between approaches.

## Discussion

DAA has been investigated as an alternative to the posterior or anterolateral approach to the hip for THA for some time. DAA has gained popularity thanks to reported shorter hospital stays, reduced dislocation rates, and reduced postoperative pain when compared to the posterior approach [5]. In this study, our primary outcome measure was patient-reported OHS at three months and one year, compared to PA and ALA.

The evidence suggests that patient-reported functional outcomes improved following DAA at three months, six months, and one year in a study involving a cohort of 523 DAA patients and 525 PA patients [5]. The outcome measure examined was the Harris Hip Score (HHS). Our study collected data for OHS, which is also a validated outcome measure in THA [19]. No significant difference was found between the approaches in our study, which has been validated in other studies. Tsukada et al. [20] also demonstrated improved early outcomes with DAA compared to the posterior approach, but non-significant improvement at later follow-ups. There is well-established evidence for a faster postoperative recovery with reduced length of stay in hospital following THA via DAA when compared to other approaches [5]. Our study found a significantly shorter length of stay in a hospital where all patients are cared for in the same environment, with the same nursing staff, physiotherapists, and occupational therapists. There is also evidence showing greater postoperative blood loss following DAA vs. PA; however, there was no significant difference in our cohort [5].

The DAA approach retains the integrity of the posterior structures (i.e. capsule, short external rotators), and hence there is a low dislocation rate. Tsukada et al. [20] reported a 0% dislocation rate in 139 patients with five-year follow-up and a 4% dislocation rate in 177 patients who underwent PA. Another study reported zero dislocations in a series of 93 DAA cases following the adoption of the approach by surgeons previously experienced in the posterior and anterolateral approach [21]. Our series presented no dislocations, periprosthetic fractures, or infections in the anterolateral, DAA, or posterior approach groups. A meta-analysis comparing complication rates between the DAA and posterior approach found comparable fracture rates with the posterior approach (0.95% vs. 1.07% with DAA) [5]. The same meta-analysis found no significant difference in infection rates and a higher dislocation rate in posterior approach THA (1.48% vs. 0.71% in DAA).

Literature review

Over the last decade, the popularity of DAA has increased as this approach preserves the hip musculature and does not breach the posterior joint capsule, minimising the risk of dislocation [4]. A meta-analysis by Chen et al. [5] compared 18 randomised and non-randomised controlled trials totalling 34,873 patients (DAA n=9636, PA n =25,237) and concluded that, when compared to PA, DAA has the benefits of a lower incidence of pulmonary embolus (PE) or deep vein thrombosis (DVT) [6], shorter hospital stay [7], lower postoperative pain [8], and a lower dislocation rate [6]. The same meta-analysis found that THA performed via DAA has a longer operative time, higher risk of intraoperative fracture, and greater blood loss when compared to PA [9]. No significant difference was found in leg length discrepancy [10], rate of infection, haematoma, or reoperation rate. The postoperative radiographic evaluation also indicated that there is no statistically significant difference in cup inclination or anteversion between DAA and PA [11]. Another study performed by Hamilton et al. [12] found less version and inclination variability across 100 DAA cases compared with posterolateral, and fluoroscopy was not used in this study.

HHS and visual analog score (VAS) for pain are frequently used in determining patient-reported outcomes following THA. There is evidence that patients have favourable VAS on days one and two post-op, following DAA vs. PA, which is reflected in reports of shorter hospital stays [8]. A meta-analysis of studies including 1048 participants has reported favourable Harris scores in DAA vs. PA at three, six, and 12 months of follow-up [13]. The soft-tissue preserving nature of the DAA combined with a low risk of dislocation has increased its popularity globally in recent times. It is muscle-splitting and has a true internervous plane with the gluteus medius and tensor fascia lata laterally, and sartorius and rectus muscles medially. This means that the risk of limping with the anterior and lateral approaches is reduced, and the higher risk of dislocation associated with posterior approaches is also avoided [14].

Although there are documented advantages to the use of minimally invasive approaches such as DAA for THA, some studies have reported poorer outcomes [15,16] and higher rates of early complication when compared to PLA. These studies have demonstrated a greater operating time and greater blood loss. There is evidence of higher early complication rates associated with DAA, particularly during the so-called learning curve [15]. Intraoperative complications and component misalignment may be more prevalent in DAA due to the constrained surgical field, making visualisation of the anatomical landmarks and alignment of the components more challenging [17]. The patient is positioned supine, facilitating the use of an image intensifier to aid implant positioning; however, this may contribute to an increase in surgical time and implant exposure. Intraoperative fracture is the most frequent complication associated with DAA.

It is not uncommon for newly adopted operations to result in a temporary increase in complications or difficulty in the learning curve. It is proposed that the learning curve for a new procedure entails 30-50 cases [17]. Concerning the adoption of DAA, Stone et al. 2018 [18] found that the learning curve was 500 cases when compared with the procedure time for the posterolateral approach. Their series of 1000 cases found that after 500 cases, the DAA operative time was shorter than posterolateral, and, at 850 cases, the DAA time was 14% shorter than posterolateral.

This study has a few limitations. Firstly, the small sample size limits the significance of the results, and further ongoing research with a greater sample size would be indicated. There was a degree of selection bias to minimise the variable which could distort the outcome measures. Patient age, ASA, and BMI were standardised in an effort to draw a direct comparison. Complete one-year follow-up data was available for 26 of 32 anterolateral patients (81%), 23 of 32 (72%) for the DAA patients, and 26 of 32 (81%) posterior approach patients, which also affects the significance of the results found.

## Conclusions

Despite the literature indicating a steep learning curve when considering the surgical time for DAA, we have found that the adoption of the approach did not lead to any significant increase in surgical time or blood loss and a significant reduction in length of stay in hospital. Despite the perceived learning curve associated with adopting this new technique, there was no significant difference in functional outcome between the approaches, as measured using OHS. We propose that DAA is a safe approach to adopt, with reduced hospital stays for patients and cost-savings associated with it. Cost-effectiveness was not explored in this study but would be a strong premise for further research.

## References

[1] Learmonth ID, Young C, Rorabeck C (2007). The operation of the century: total hip replacement. Lancet.

[2] Laupacis A, Bourne R, Rorabeck C (1993). The effect of elective total hip replacement on health-related quality of life. J Bone Joint Surg Am.

[3] Waddell J, Johnson K, Hein W, Raabe J, FitzGerald G, Turibio F (2010). Orthopaedic practice in total hip arthroplasty and total knee arthroplasty: results from the Global Orthopaedic Registry (GLORY). Am J Orthop (Belle Mead NJ).

[4] Kennon RE, Keggi JM, Wetmore RS, Zatorski LE, Huo MH, Keggi KJ (2003). Total hip arthroplasty through a minimally invasive anterior surgical approach. J Bone Joint Surg Am.

[5] Chen W, Sun JN, Zhang Y, Zhang Y, Chen XY, Feng S (2020). Direct anterior versus posterolateral approaches for clinical outcomes after total hip arthroplasty: a systematic review and meta-analysis. J Orthop Surg Res.

[6] Siljander MP, Whaley JD, Koueiter DM, Alsaleh M, Karadsheh MS (2020). Length of stay, discharge disposition, and 90-day complications and revisions following primary total hip arthroplasty: a comparison of the direct anterior, posterolateral, and direct superior approaches. J Arthroplasty.

[7] Triantafyllopoulos GK, Memtsoudis SG, Wang H, Ma Y, Alexiades MM, Poultsides LA (2019). Surgical approach does not affect deep infection rate after primary total hip arthroplasty. Hip Int.

[8] Zhao HY, Kang PD, Xia YY, Shi XJ, Nie Y, Pei FX (2017). Comparison of early functional recovery after total hip arthroplasty using a direct anterior or posterolateral approach:a randomized controlled trial. J Arthroplasty.

[9] Barrett WP, Turner SE, Leopold JP (2013). Prospective randomized study of direct anterior vs postero-lateral approach for total hip arthroplasty. J Arthroplasty.

[10] Godoy-Monzon D, Buttaro M, Comba F, Piccaluga F, Cid-Casteulani A, Ordas A (2019). Comparative study of radiological and functional outcomes following a direct anterior approach versus to a posterolateral approach to the hip. Rev Esp Cir Ortop Traumatol (Engl Ed).

[11] Nam D, Sculco PK, Abdel MP, Alexiades MM, Figgie MP, Mayman DJ (2013). Leg-length inequalities following THA based on surgical technique. Orthopedics.

[12] Hamilton WG, Parks NL, Huynh C (2015). Comparison of cup alignment, jump distance, and complications in consecutive series of anterior approach and posterior approach total hip arthroplasty. J Arthroplasty.

[13] Barrett WP, Turner SE, Murphy JA, Flener JL, Alton TB (2019). Prospective, randomized study of direct anterior approach vs posterolateral approach total hip arthroplasty: a concise 5-year follow-up evaluation. J Arthroplasty.

[14] Bender B, Nogler M, Hozack WJ (2009). Direct anterior approach for total hip arthroplasty. Orthop Clin North Am.

[15] D'Arrigo C, Speranza A, Monaco E, Carcangiu A, Ferretti A (2009). Learning curve in tissue sparing total hip replacement: comparison between different approaches. J Orthop Traumatol.

[16] Woolson ST, Pouliot MA, Huddleston JI (2009). Primary total hip arthroplasty using an anterior approach and a fracture table: short-term results from a community hospital. J Arthroplasty.

[17] Spaans AJ, van den Hout JA, Bolder SB (2012). High complication rate in the early experience of minimally invasive total hip arthroplasty by the direct anterior approach. Acta Orthop.

[18] Stone AH, Sibia US, Atkinson R, Turner TR, King PJ (2018). Evaluation of the learning curve when transitioning from posterolateral to direct anterior hip arthroplasty: a consecutive series of 1000 cases. J Arthroplasty.

[19] Murray DW, Fitzpatrick R, Rogers K, Pandit H, Beard DJ, Carr AJ, Dawson J (2007). The use of the Oxford hip and knee scores. J Bone Joint Surg Br.

[20] Tsukada S, Wakui M (2015). Lower dislocation rate following total hip arthroplasty via direct anterior approach than via posterior approach: five-year-average follow-up results. Open Orthop J.

[21] Khatod M, Barber T, Paxton E, Namba R, Fithian D (2006). An analysis of the risk of hip dislocation with a contemporary total joint registry. Clin Orthop Relat Res.

